# Supplementary material for: Coproducing a physical activity referral scheme in Germany: a qualitative analysis of stakeholder experiences
Source: BMJ Open. 2024 May 22;14(5):e082710. doi: 10.1136/bmjopen-2023-082710 (PMC11116878; doi:10.1136/bmjopen-2023-082710)
Supplement: Supplementary data [file bmjopen-2023-082710supp001.pdf]

Project *BewegtVersorgt*: Evaluation of the co-production process (1. and 2. project phase)
Interview guide for semi-structured interviews (via video call)
Version 04 May 2022

Introduction
(Welcome, research subject, note on privacy policy)

**Guiding question 1 (Motivation): How did you get involved in the project *BewegtVersorgt*?**			
Topic	***Probing questions***	***Specific questions***	***Notes***
Motivation, goals	Is there anything else you would like to share on this topic?<br><br>Can you talk more about that?<br><br>Would you explain that in more detail?<br><br>What exactly do you mean by that?<br><br>Is there anything else you can think of?<br><br>Do you have any other thoughts on this topic?	Who or what motivated you to participate?	
		Which **goals** did you pursue with your participation?<br><br>-> If necessary, ask: goals of the organisation	

<table>
<tr><td colspan="3">Guiding question 2 (Co-production process):
a) What was your experience with the joint co-production meetings in the first project phase?
b) In the BewegtVersorgt project, we used a participatory approach bringing various stakeholders from the healthcare sector around the same table. How would you describe your experience with this participation approach?</td><td></td></tr>
<tr><td>Topic</td><td>Probing questions</td><td>Specific questions</td><td>Notes</td></tr>
<tr><td>Group composition</td><td rowspan="4">Is there anything else you would like to share on this topic?

Can you talk more about that?

Would you explain that in more detail?

What exactly do you mean by that?

Is there anything else you can think of?

Do you have any other thoughts on this topic?</td><td>How do you evaluate the group composition - Should more or fewer different stakeholders have participated in the co-production of the PARS?

-> If necessary, ask: Which actors were missing?</td><td></td></tr>
<tr><td>Representation/ participation</td><td>How would you describe your role in the co-production process?

-> If necessary, ask: What was your role compared to other partners?</td><td></td></tr>
<tr><td>Collaboration</td><td>How did you feel about the collaboration with the other partners?

-> If necessary, ask: Reasons for problems in collaboration.</td><td></td></tr>
<tr><td>Appropriateness of the co-production process</td><td>When you think about the "agreement" on the final PARS, what role did the joint co-production process have?

-> If necessary, ask: How do you assess the relationship between effort and benefit in this process? (Could we have reached the goal easier?)</td><td></td></tr>
</table>

		If you had the opportunity to repeat the PARS co-production process, what should be done differently from your perspective?	
Management/ Coordination		The BewegtVersorgt project team organised the co-production process and led the individual co-production meetings. How did you feel about the steering role of the project team?	

**Guiding question 3 (The result): Finally, I would like to talk to you about the result of this joint co-development phase. How do you evaluate the developed Physical Activity Referral Scheme (PARS)?**			
Topic	***Probing questions***	***Specific questions***	***Notes***
Suitability of the PARS	Is there anything else you would like to share on this topic?<br><br>Can you talk more about that?<br><br>Would you explain that in more detail?<br><br>What exactly do you mean by that?<br><br>Is there anything else you can think of?<br><br>Do you have any other thoughts on this topic?	What do you think, how suitable is the developed PARS for getting people with **non-communicable chronic diseases** to increase their physical activity levels in the long term?<br><br>-> If necessary, ask for adaptations of the PARS.	
Role/task distribution within PARS		In the co-produced PARS, the individual professional groups (scheme deliverers) take on different tasks and roles. How do you assess the distribution of roles with the PARS?<br><br>-> If necessary, ask for adaptations of role distribution.	
PARS transfer and scaling up		The developed PARS will be integrated into the healthcare system if proven effective. What do you think is important for this **integration to be sustainable**?	

Interview closure
(Announce the end of the interview, ask if there is anything left to tell/unspoken thoughts, and thank the interviewee for participation.)
